# Serum CETP status is independently associated with reduction rates in LDL-C in pitavastatin-treated diabetic patients and possible involvement of LXR in its association

**DOI:** 10.1186/s12944-016-0223-6

**Published:** 2016-03-17

**Authors:** Akihiro Shimada, Hideki Kimura, Koji Oida, Hideo Kanehara, Yukihiro Bando, Shinobu Sakamoto, Takanobu Wakasugi, Takashi Saga, Yasuki Ito, Kazuko Kamiyama, Daisuke Mikami, Masayuki Iwano, Tsutomu Hirano, Haruyoshi Yoshida

**Affiliations:** Department of Clinical Laboratory and Nephrology, University of Fukui Hospital, 23-3 Matsuoka-shimoaizuki, Eiheiji, Yoshida, Fukui 910-1193 Japan; Division of Internal Medicine, Fukui Chuo Clinic, 4-5-10, Matsumoto, Fukui 910-0003 Japan; Division of Endocrinology, Fukui-ken Saiseikai Hospital, 7-1, Funahashi, Wadanaka, 918-8503 Japan; Division of Internal Medicine, Yasukawa Hospital, 2-108, Owada, Fukui 910-0005 Japan; Division of Endocrinology, Fukui Prefectural Hospital, 2-8-1, Yotsui, Fukui 910-8526 Japan; Division of Internal Medicine, Tanaka Hospital, 2-3-1 Ohte, Fukui, Fukui 910-0005 Japan; Research and Development Department, Denka Seiken Co. Ltd, Tokyo, Japan; Division of Nephrology, Department of Medicine, School of Medicine, Faculty of Medical Sciences, University of Fukui, 23-3 Matsuoka-shimoaizuki, Eiheiji, Yoshida, Fukui 910-1193 Japan; Department of Medicine, Division of Diabetes, Metabolism, and Endocrinology, Showa University School of Medicine, Tokyo, Japan; Division of Nephrology, Obama Municipal Hospital, 2-2 Ohte, Obama, Fukui 917-8567 Japan

**Keywords:** CETP, Pitavastatin, Liver X receptor, T0901317, Small dense LDL-C

## Abstract

**Background:**

Statins decrease cholesteryl ester transfer protein (CETP) levels, which have been positively associated with hepatic lipid content as well as serum low density lipoproteins-cholesterol (LDL-C) levels. However, the relationship between the CETP status and statin-induced reductions in LDL-C levels has not yet been elucidated in detail. We herein examined the influence of the CETP status on the lipid-reducing effects of pitavastatin in hypercholesterolemic patients with type 2 diabetes mellitus as well as the molecular mechanism underlying pitavastatin-induced modifications in CETP levels.

**Methods:**

Fifty-three patients were treated with 2 mg of pitavastatin for 3 months. Serum levels of LDL-C, small dense (sd) LDL-C, and CETP were measured before and after the pitavastatin treatment. The effects of pitavastatin, T0901317, a specific agonist for liver X receptor (LXR) that reflects hepatic cholesterol contents, and LXR silencing on CETP mRNA expression in HepG2 cells were also examined by a real-time PCR assay.

**Results:**

The pitavastatin treatment decreased LDL-C, sdLDL-C, and CETP levels by 39, 42, and 23 %, respectively. Despite the absence of a significant association between CETP and LDL-C levels at baseline, baseline CETP levels and its percentage change were an independent positive determinant for the changes observed in LDL-C and sdLDL-C levels. The LXR activation with T0901317 (0.5 μM), an in vitro condition analogous to hepatic cholesterol accumulation, increased CETP mRNA levels in HepG2 cells by approximately 220 %, while LXR silencing markedly diminished the increased expression of CETP. Pitavastatin (5 μM) decreased basal CETP mRNA levels by 21 %, and this was completely reversed by T0901317.

**Conclusion:**

Baseline CETP levels may predict the lipid-reducing effects of pitavastatin. Pitavastatin-induced CETP reductions may be partially attributed to decreased LXR activity, predictable by the ensuing decline in hepatic cholesterol synthesis.

**Trial registration:**

UMIN Clinical Trials Registry ID UMIN000019020

## Background

Cholesteryl ester transfer protein (CETP) is a 74-kDa glycoprotein that is synthesized in liver and adipose tissues and subsequently secreted into the bloodstream [1]. Circulating CETP transfers the cholesterol ester (CE) from low density lipoproteins (LDL) and high density lipoproteins (HDL) to very low density lipoproteins (VLDL) in an equimolecular exchange of triglycerides (TG) according to their concentrations [2]. Consequently, CETP levels are positively associated with LDL-C levels [3, 4] and negatively associated with HDL-C levels [3, 4]. The expression of CETP was recently reported to be induced by the liver X receptor (LXR) [5, 6]. As LXR has been identified as a hepatic cholesterol sensor [7], serum CETP levels may also be a surrogate for hepatic lipid content [8].

Small dense (sd) LDL is known to be more atherogenic than LDL due to its longer half-time, greater penetrance into arterial walls, and greater susceptibility to oxidative stress [9]. Several clinical studies demonstrated that smaller LDL particles were associated with the incidence of coronary artery disease [10–12]. Dyslipidemia in type 2 diabetes mellitus is characterized by high TG levels, low HDL-C levels, and high sd LDL –C levels [13]. In type 2 diabetes mellitus, the production of TG-enriched VLDL is increased, which readily causes the formation of TG-rich LDL, a precursor of sd LDL [13, 14]. In the diabetic condition, CETP also enhances the production of TG-rich LDL due to its functional properties, leading to preferable formation of sd LDL [2, 13].

HMG-CoA reductase inhibitors (statins) are widely used to treat hypercholesterolemia and are known to be useful for reducing cardiovascular disease events. Previous studies reported that some statins such as pitavastatin markedly decreased sd LDL-C as well as LDL-C, [15, 16] and also significantly reduced circulating CETP levels [4, 17]. Considering that CETP plays some role in the production of atherogenic lipid profiles, its status may modify lipid-reducing effects of statin treatment. However, this remains to be clarified. The aim of the present study, therefore, was to assess not only the influence of the CETP status on the lipid-reducing effects of pitavastatin in type 2 diabetic patients with hypercholesterolemia, but also the molecular mechanism underlying pitavastatin-induced modifications in the expression of CETP in HepG2 cell lines.

## Results

### Baseline characteristics

The backgrounds of 53 hypercholesterolemic patients with type 2 diabetes are shown in Table 1. The mean age of the patients was 61.1 ± 9.8 years. Twenty-seven patients were male and 26 were female. The means of body mass index and HbA1c were 24.8 ± 4.5 kg/m^2^ and 7.1 ± 0.8 % (NGSP), respectively. Regarding diabetes-related complications, 5 had diabetic nephropathy (9.4 %). Regarding cardiovascular diseases, 23 patients had hypertension (43.4 %), and 4 had coronary artery disease (7.5 %).

**Table 1:** The background of the enrolled patients

Characteristics	*N* = 53
Age (years)	61.1 ± 9.8^a^
Male, *n* (%)	27 (50.9)^b^
Body mass index (kg/m^2^) *n* = 52	24.8 ± 4.5
Abdominal girth (cm) *n* = 48	88.7 ± 9.4
Systolic blood pressure (mmHg) *n* = 52	127.9 ± 12.0
Diastolic blood pressure (mmHg) *n* = 52	73.3 ± 9.2
HbA1c (%)	7.1 ± 0.8
Complications	
Type 2 diabetes, *n* (%)	53 (100.0)^b^
diabetic neuropathy, *n* (%)	10 (18.9)
diabetic nephropathy, *n* (%)	5 (9.4)
diabetic retinopathy, *n* (%)	4 (7.5)
Hypertension, *n* (%)	23 (43.4)
Coronary artery disease, *n* (%)	4 (7.5)
Cerebral artery disease, *n* (%)	1 (1.9)
Peripheral artery disease, *n* (%)	2 (3.8)
Concurrent medications	
Anti-diabetic agents	
sulfonyl urea	28 (52.8)^b^
biguanide	25 (47.2)
α-glucosidase inhibitor	23 (43.4)
pioglitazone	10 (18.9)
Insulin	5 (9.4)
Anti-hypertensive agents	
angiotensin II type 1 receptor blockers (ARBs)	18 (34.0)^b^
calcium channel blockers	13 (24.5)
diuretics	7 (13.2)
angiotensin-converting enzyme inhibitors	5 (9.4)
Anti-lipidemic agents	
fenofibrate	2 (3.8)^b^

^a^Data present mean ± SD

^b^Data present the number and its percentage (%)

The concurrent administration of drugs for these subjects is shown in Table 1. As for anti-diabetes agents, sulfonyl ureas were prescribed for 28 patients, biguanides for 25, α-glucosidase inhibitors for 23, pioglitazone for 10, and insulin for 5. The anti-lipidemic agent, fenofibrate was prescribed for two patients only.

### Changes in clinical laboratory data

The changes observed in clinical laboratory data are shown in Table 2. The pitavastatin treatment significantly decreased total cholesterol (TC), LDL-C, and sd-LDL-C serum levels by 25.7, 39.4, and 41.7 %, respectively. Pitavastatin also significantly decreased sd LDL-C/LDL-C levels by 4.5 % and serum CETP levels by 22.9 %, and significantly increased ApoA-I levels by 3.5 %. In contrast, no significant changes were observed in TG, HDL-C, or HbA1c levels.

**Table 2:** Clinical data profile of pre- and post-pitavastatin treatment

Variables	*n*	baseline	*n*	Post-treatment	Change (%)	*P* value
Body weight (kg)	53	64.1 ± 15.4	52	64.4 ± 16.2	0.7 ± 2.4	0.052
HbAlc (%)	53	7.1 ± 0.80	53	7.2 ± 0.84	1.6 ± 7.6	0.13
eGFR (ml/min/m^2^)	52	75.4 ± 16.9	51	76.8 ± 16.5	4.2 ± 17.2	0.18
BUN (mg/dL)	52	14.7 ± 4.2	50	14.8 ± 3.9	−2.3 ± 23.8	0.49
Uric acid (mg/dL)	52	5.2 ± 1.4	51	4.9 ± 1.2	−4.7 ± 14.1	0.02
TC (mg/dL)	53	234.3 ± 28.9	48	173.0 ± 25.8	−25.7 ± 9.9	<0.01
TG (mg/dL)	53	146.7 ± 74.1	48	134.2 ± 87.0	−4.1 ± 55.3	0.61
HDL-C (mg/dL)	53	58.8 ± 14.8	49	58.7 ± 15.5	−0.6 ± 11.4	0.7
LDL-C (mg/dL)	53	152.1 ± 29.2	49	91.2 ± 25.4	−39.4 ± 13.6	<0.01
sd LDL-C (mg/dL)	53	52.0 ± 18.1	49	28.7 ± 10.4	−41.7 ± 18.0	<0.01
sd LDL-C/LDL-C (%)	53	33.9 ± 8.4	49	31.6 ± 7.3	−4.5 ± 15.6	0.047
ApoA-I (mg/dL)	53	142.9 ± 26.6	48	148.5 ± 26.7	3.5 ± 8.4	<0.01
CETP (μg/mL)	48	2.54 ± 0.60	46	1.98 ± 0.73	−22.9 ± 19.5	< 0.01

### Relationships between clinical factors and LDL-C, sd-LDL-C, or CETP levels at baseline

At baseline, LDL-C levels were negatively correlated with age and ApoA-I levels (*r* = −0.343, *P* < 0.05 and *r* = −0.321, *P* < 0.05, respectively) and positively associated with CETP levels (*r* = 0.184, *P* = 0.21). A marginally positive association was observed between LDL-C and CETP levels (*r* = 0.284, *P* < 0.06) in patients with a CETP value of less than 4.0 μg/mL (*n* = 47). sd LDL-C levels were positively correlated with TG levels (*r* = 0.629, *P* < 0.01), but not with CETP levels.

CETP levels were negatively correlated with age (*r* = −0.347, *P* < 0.01), but were not associated with body weight or abdominal girth. Furthermore, patients receiving pioglitazone (*n* = 10) had a significantly lower level of CETP than those receiving no pioglitazone (*n* = 38; 2.12 ± 0.48 vs. 2.66 ± 0.58 μg/mL, *P* < 0.05) after adjustments were made for age, while no other drugs had any influence on CETP levels. Since pioglitazone use was a reducer of CETP levels, it was included as an independent variable when associations of CETP status with percent changes in LDL-C and sdLDL-C levels were analyzed.

### Relationship between clinical variables and percentage changes in LDL-C

We analyzed relationships between the baseline levels of clinical variables and percentage changes in LDL-C levels to determine what clinical variables affected pitavastatin-induced changes in LDL-C levels (Table 3). Selected candidate determinants for changes in LDL-C were shown in Table 3. Multiple regression analyses identified TC as an independent negative determinant and CETP as an independent positive determinant (Table 3). When four patients receiving previous treatment with mild statin were excluded, the same results were obtained. CETP failed to be an independent predictor when the two patients with a CETP level of 4.9 μg/mL were included.

**Table 3:** Linear regression analyses of baseline values and percent changes in clinical factors affecting the percent change in LDL-C levels

	Univariate				Multivariate			
	*n*	β^a^	95 % CI^b^	*p*	*n*	β^c^	95 % CI^b^	*p*
BMI (kg/m^2^)	46	0.17	−0.39 ~ 1.39	0.27^d^				
Gender (Male = 1, Female = 2)	47	−0.13	−11.5 ~ 4.59	0.39				
Age (year)	47	−0.24	−0.72 ~ 0.06	<0. 1	44	−0.07	−0.55 ~ 0.38	0.7
Body weight (kg)	47	0.3	0.02 ~ 0.52	0.04	44	0.07	−0.24 ~ 0.36	0.68
Abdominal girth (cm)	45	0.11	−0.28 ~ 0.59	0.47				
HbA1c (%)	47	0.08	−3.57 ~ 6.38	0.57				
Uric acid (mg/dL)	47	0.05	−2.3 ~ 3.26	0.74				
TC (mg/dL)	47	−0.37	−0.29 ~ −0.04	0.011	44	-0.39	−0.30 ~ −0.05	0.009
LDL-C (mg/dL)	47	−0.21	−0.22 ~ 0.04	0.15^e^				
TG (mg/dL)	47	−0.1	−0.07 ~ 0.04	0.5				
HDL-C (mg/dL)	47	−0.32	−0.56 ~ −0.03	0.03	44	−0.16	−0.42 ~ 0.13	0.29
sd LDL-C (mg/dL)	47	−0.29	−0.43 ~ 0.01	0.04^e^				
ApoA-I (mg/dL)	47	−0.28	−0.29 ~ 0.003	0.05^f^				
CETP (μg/mL)	44	0.38	2.09 ~ 15.69	0.01	44	0.41	2.91 ~ 16.5	0.006
Pioglitazone use	47	−0.02	−12.1 ~ 10.63	0.9				
Change in Body weight (%)	47	0.19	−0.57 ~ 2.66	0.2	43	0.36	0.44 ~ 3.64	0.01
Change in HbA1c (%)	47	−0.009	−0.56 ~ 0.52	0.95				
Change in Uric acid (%)	47	0.14	−0.15 ~ 0.43	0.33				
Change in TG (%)	46	0.22	−0.02 ~ 0.12	0.15	43	0.17	−0.02 ~ 0.10	0.2
Change in HDL-C (%)	47	−0.07	−0.47 ~ 0.29	0.64				
Change in ApoA-I (%)	46	0.06	−0.48 ~ 0.71	0.71				
Change in CETP (%)	44	0.44	0.12 ~ 0.52	0.003	43	0.57	0.21 ~ 0.60	0.0001

^a^standard regression coefficient

^b^confidence interval

^c^standard partial regression coefficient

^d^BMI was excluded from multivariate analysis, since colinearity between BMI and body weight was found

^e^LDL-C and sd LDL-C were excluded from multivariate analysis, since colinearity among TC, LDL-C and sd LDL-C was found

^f^ApoA-I was excluded from multivariate analysis, since colinearity between HDL-C and ApoA-I was found

Relationships between percentage changes in clinical variables and changes in LDL-C levels were then analyzed (Table 3). Selected candidate determinants for changes in LDL-C were shown in Table 3. A multiple regression analysis retained changes in body weight and CETP as independent positive determinants, with the standard partial correlation coefficient of the latter being markedly stronger (Table 3). When the two patients with a CETP level of 4.9 μg/mL were included or four patients with previous statin treatment were excluded, the multiple regression analysis produced the same results.

Furthermore, percentage reductions in LDL-C levels were significantly larger in the subgroup with sub-median CETP levels at baseline (<2.6 μg/mL, *n* = 21) than in the groups with supra-median CETP values (*n* = 23; Table 4). Similar results were also found when the two patients with a CETP level of 4.9 μg/mL were included.

**Table 4:** Comparison of lipid data between patients with below- and abovemedian CETP levels

	Patients with CETP levels below the median (<2.6μg/mL; *n* = 21 )	Patients with CETP levels above the median (≧ 2.6 μg/mL; *n* = 23 )	*p*
Baseline CETP (p,g/mL)	2.12 ± 0.3	3.01 ± 0.44	<0.01
Post-treatment CETP (p,g/mL)	1.48 ± 0.36	2.44 ± 0.71	<0.01
Change in CETP (%)	−28.9 ± 20.3	−19.2 ± 17.0	0.09
Baseline LDL-C (mg/dL)	143.5 ± 234.1	157.0 ± 34.9	0.14
Post-treatment LDL-C (mg/dL)	75.1 ± 15.2	103.6 ± 26.3	<0.01
Change in LDL-C (%)	−46.4 ± 11.2	−33.1 ± 13.1	<0.01
Baseline sd LDL-C (mg/dL)	51.8 ± 16.3	51.7 ± 20.6	0.98
Post-treatment sd LDL-C (mg/dL)	23.9 ± 6.5	32.7 ± 12.1	<0.01
Change in LDL-C (%)	−49.6 ± 13.7	−33.5 ± 19.2	<0.01

### Relationship between clinical variables and percentage changes in sd LDL-C

We analyzed relationships between the baseline levels of clinical variables and pitavastatin-induced changes in sd LDL-C levels (Table 5). Selected candidate determinants for percentage changes in sd LDL-C levels were shown in Table 5. Multiple regression analyses retained TC as an independent negative determinant and CETP as an independent positive determinant (Table 5). Exclusion of four patients with previous statin treatment produced the same results. When the two patients with a CETP level of 4.9 μg/mL were included, CETP failed to be an independent determinant.

**Table 5:** Linear regression analyses of baseline values and percent changes in clinical factors affecting the percent change in sd LDL-C levels

	Univariate				Multivariate			
	*n*	β^a^	95 % CI^b^	*p*	*n*	β^c^	95 % CI^b^	*p*
BMI (kg/m^2^)	46	0.04	−1.07 ~ 1.37	0.8				
Gender (Male = 1, Female = 2)	47	−0.18	−17.18 ~ 4.34	0.24	44	−0.11	−16.4 ~ 8.13	0.5
Age (year)	47	−0.1	−0.72 ~ 0.36	0.5				
Body weight (kg)	47	0.18	−0.13 ~ 0.56	0.22	44	0.03	−0.38 ~ 0.44	0.88
Abdominal girth (cm)	45	0.08	−0.44 ~ 0.74	0.61				
HbA1c (%)	47	0.2	−2.07 ~ 11.1	0.17	44	0.19	−2.54 ~ 11.1	0.21
Uric acid (mg/dL)	47	0.1	−2.45 ~ 5.02	0.49				
TC (mg/dL)	47	−0.42	−0.41 ~ −0.09	0.004	44	−0.43	−0.46 ~ −0.06	0.01
LDL-C (mg/dL)	47	-0.29	−0.34 ~ −0.002	0.05^d^				
TG (mg/dL)	47	−0.18	−0.12 ~ 0.03	0.23	44	−0.04	−0.09 ~ 0.07	0.8
HDL-C (mg/dL)	47	−0.24	−0.66 ~ 0.06	0.11	44	−0.02	−0.45 ~ 0.41	0.92
sd LDL-C (mg/dL)	47	−0.42	−0.68 ~ −0.14	0.004^d^				
ApoA-I (mg/dL)	47	−0.22	−0.36 ~ 0.05	0.14^e^				
CETP (μg/mL)	44	0.29	−0.15 ~ 18.67	0.054	44	0.32	0.27 ~ 20.2	0.04
Pioglitazone use	47	0.02	−14.13 ~ 16.5	0.88				
Change in Body weight (%)	47	0.35	0.05 ~ 4.29	0.02	43	0.43	1.24 ~ 5.26	0.002
Change in HbA1c (%)	47	−0.13	−1.2 ~ 0.23	0.37				
Change in Uric acid (%)	47	0.12	−0.22 ~ 0.56	0.41				
Change in TG (%)	46	0.32	0.01 ~ 0.2	0.03	43	0.26	−0.0001 ~ 0.16	0.05
Change in HDL-C (%)	47	0.011	−0.42 ~ 0.61	0.94				
Change in ApoA-I (%)	46	0.11	−0.31 ~ 1.28	0.48				
Change in CETP (%)	44	0.32	0.03 ~ 0.6	0.03	43	0.46	0.19 ~ 0.66	0.001

^a^standard regression coefficient

^b^confidence interval

^c^standard partial regression coefficient

^d^LDL-C and sd LDL-C were excluded from multivariate analysis, since colinearity among TC, LDL-C and sd LDL-C was found

^e^ApoA-I was excluded from multivariate analysis, since colinearity between HDL-C and ApoA-I was found

Relationships between percentage changes in clinical variables and changes in sd LDL-C were then analyzed (Table 5). Selected candidate determinants for the changes induced in sd LDL-C were shown in Table 5. A multiple regression analysis retained changes in body weight, and CETP as independent positive determinants, with the standard partial correlation coefficient of the last being the strongest (Table 5). When the two patients with a CETP level of 4.9 μg/mL were included or four patients with previous statin treatment were excluded, the multiple regression analysis produced the same results.

Furthermore, percentage reductions in sd LDL-C levels were significantly greater in the subgroup with sub-median CETP levels than in the subgroup with supra-median CETP levels (Table 4). Similar results were also observed when the two patients with a CETP level of 4.9 μg/mL were included.

### Effects of pitavastatin and T0901317 on the expression of lipid metabolism-related genes in HepG2 cells

First, we examined the cytotoxic effects of pioglitazone and T0901317 on HepG2 cells by measuring LDH levels in the supernatants. Pitavastatin did not significantly increase the levels of LDH in the supernatants at 1, 2.5 or 5 μM (109 ± 22 %, 103 ± 15 %, and 110 ± 17 %, respectively). T0901317 did not significantly increase the LDH levels at 0.1, 1 or 10 μM (94 ± 4 %, 97 ± 25 %, and 101 ± 1 % and 92 ± 11 %, respectively). Therefore, pitavastatin and T0901317 are considered to have no cytotoxic effects at the above concentrations.

**Fig. 1:**
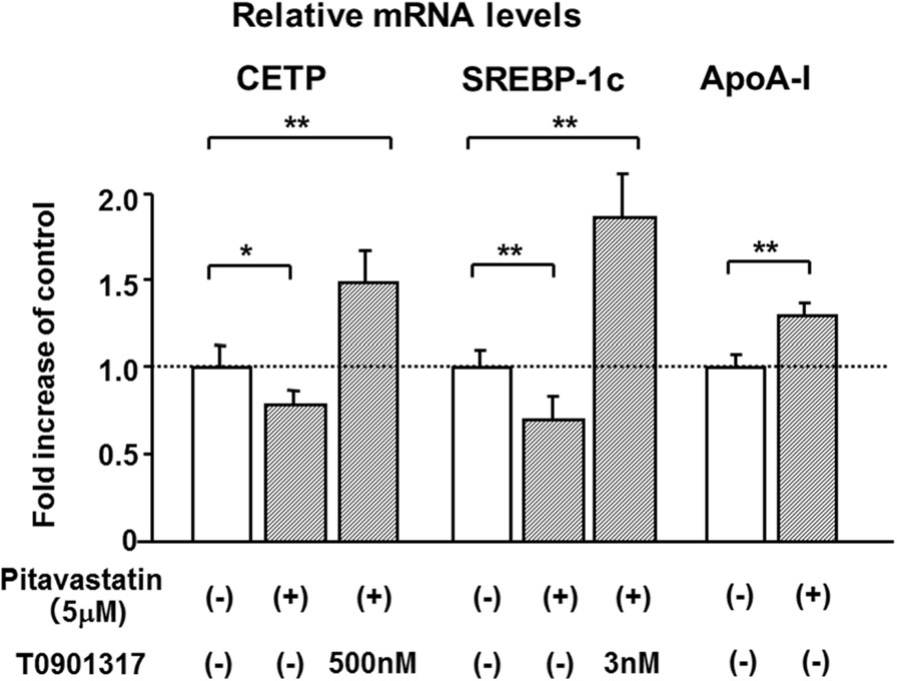
Pitavastatin decreased CETP and SREBP-1c mRNA levels, and increased ApoA-I mRNA levels in HepG2 cells, and T0901317 abolished the decreasing effects. HepG2 cells grown to semi-confluence were treated with DMEM containing no pitavastatin or T0901317 (control), 5 μM pitavastatin alone, or 5 μM pitavastatin plus T0901317 (3 or 500 nM) for 24 h. Results were the mean ± SD of three independent experiments in duplicate or triplicate (*n* = 8-9). *P < 0.05 and **P < 0.01 significantly different from cells under the indicated conditions, according to ANOVA with Scheffé’s post hoc test. Footnote: Concentrations used of T0901317 were different (500nM for CETP and 3 nM for SREBP-1c) in order to show clearly significant differences in CETP and SREBP-1c expression among three groups

**Fig. 2:**
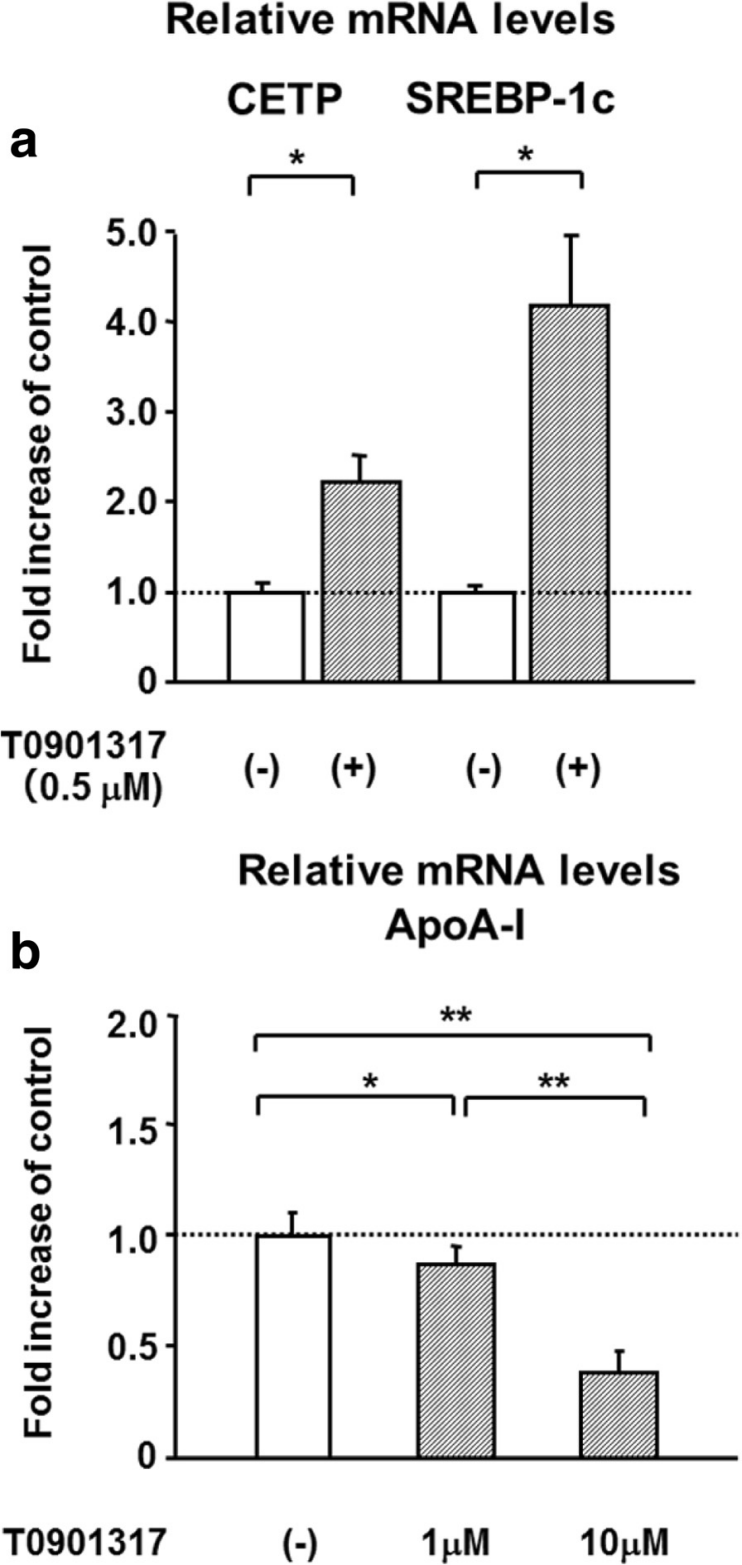
T0901317 increased CETP and SREBP-1c mRNA levels, and decreased ApoA-I mRNA levels in HepG2 cells. HepG2 cells grown to semi-confluence were treated with DMEM containing 0 (control), 1, or 10 μM T0901317 for 24 h. TaqMan real-time PCR was performed for CETP (**a**), SREBP-1c (**a**), ApoA-I (**b**), and β-actin. Results were the mean ± SD of three to five independent experiments in triplicate (*n* = 9–15). *P < 0.05 and **P < 0.01 significantly different from cells under the indicated conditions, according to an unpaired *t*-test for Fig. 2a and ANOVA with Scheffé’s post hoc test for Fig. 2b Footnote: The inhibitory effect of T0901317 on ApoA-I expression was relatively weak and thus the concentrations of 1 and 10 μM were used in order to show a considerable reduction of ApoA-I expression

**Fig. 3:**
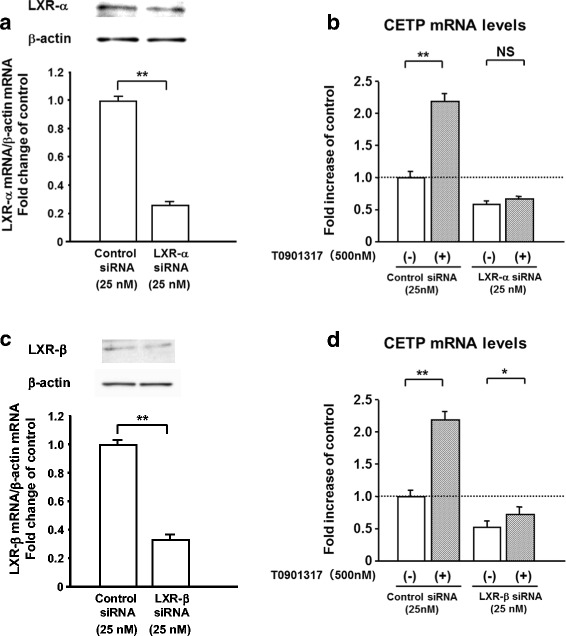
LXR silencing inhibited T0901317-stimulated CETP expression in HepG2 cells. HepG2 cells (30–40 % confluence) were transfected with negative control siRNA or siRNA against LXR-α (**a** and **b**) or LXR-β (**c** and **d**) (25nM each). After a 48-h incubation, HepG2 cells that had been refreshed with DMEM were treated with or without T0901317 (500 nM) for an additional 24 h (**b** and **d**). Protein amounts of LXR-α (**a**), LXR-β (**c**), and β-actin in the cell lysates were determined by immunoblot analyses. Results were the mean ± SD of an independent experiment in triplicate or quadruplicate (*n* = 3-4). NS, not significant; *P < 0.05 and **P < 0.01 significantly different from cells under the indicated conditions, according to an unpaired *t*-test for Fig. 3a and c and ANOVA with Scheffé’s post hoc test for Fig. 3b and d

Pitavastatin (5 μM) decreased CETP and sterol regulatory element-binding protein-1c (SREBP-1c) mRNA levels by 21 and 30 % at 5 μM, respectively (Fig. 1), and increased ApoA-I mRNA levels by 29 % (Fig. 1). After pitavastatin treatment (1 and 5 μM), mRNA levels of LXR-α and LXR-β did not change significantly (97 ± 6 % and 107 ± 10 %, and 101 ± 1 % and 102 ± 9 %, respectively). T0901317, a specific agonist of LXR-α and LXR-β, significantly elevated CETP mRNA levels by 1.8-fold at 0.1 μM, by 2.2-fold at 0.5 μM, by 2.2-fold at 1.0 μM, and by 2.3-fold at 10 μM and SREBP-1c mRNA levels by 1.8-fold at 3 nM, by 2.7-fold at 10 nM, by 3.7-fold at 100 nM, by 4.2-fold at 0.5 μM, by 4.0-fold at 1.0 μM, and by 4.0-fold at 10 μM, reaching a plateau of 2.2- and 4.2-fold at 0.5 μM (Fig. 2a), respectively, while it decreased ApoA-I mRNA levels by 11 and 61 % at 1 and 10 μM, respectively (Fig. 2b). T0901317 also reversed the pitavastatin-induced decline in the gene expression levels of CETP and SREBP-1c (Fig. 1). The reduction observed in the expression of SREBP-1c was completely abolished by T0901317, even at a concentration as low as 3 nM (Fig. 1) as well as 0.5 μM. Furthermore, the silencing of LXR-α and LXR–β markedly inhibited the T0901317-induced expression of CETP mRNA (Fig. 3). These results confirmed that CETP and SREBP-1c were positive target genes of LXR in HepG2 cells, as reported previously [6, 7].

## Discussion

Sd LDL has been shown to be more atherogenic than LDL due to its longer half-time, greater penetrance into arterial walls, and susceptibility to oxidative stress [9]. Several clinical studies reported that smaller LDL particles were associated with the incidence of coronary artery disease [10–12]. Dyslipidemia in type 2 diabetes mellitus is characterized by high TG levels, low HDL-C levels, and high sd LDL levels [13]. In type 2 diabetes mellitus, free fatty acids, which are released from adipocytes due to insulin resistance, are taken up by the liver. The resulting hepatic accumulation of free fatty acids increases TG-enriched VLDL production and readily gives rise to the formation of TG-rich LDL. The hepatic lipase-processing of TG-rich LDL finally produces small dense LDL particles. These mechanisms account for the increase frequently observed in sd LDL levels in type 2 diabetes mellitus [13, 14]. The greater atherogenic feature of sd LDL than LDL appears to be of importance when evaluating the reducing effects of statin treatments on sd LDL-C as well as LDL-C levels in hypercholesterolemic patients with type 2 diabetes mellitus.

In the present study, pitavastatin produced a marked reduction not only in LDL-C, but also sd LDL-C levels. This result supported earlier clinical studies in which statin reduced sd LDL-C levels in patients with diabetes mellitus [15] or familial hypercholesterolemia [16]. Both LDL reductions were largely explained by the increased expression of LDL receptors due to the decreased synthesis of intrahepatic cholesterol. Sd LDL particles have been shown to have a weaker affinity for the LDL receptor than large buoyant LDL [18], while the induction of the LDL receptor by statin reportedly stimulates the uptake of all LDL particles, irrespective of their size [15]. Thus, the pitavastatin-induced expression of LDL receptors may have enhanced the hepatic uptake of sd LDL in addition to LDL, which subsequently led to reductions in both LDL-C and sd LDL-C levels.

One of the most important results of the present study was the correlation between serum CETP levels and the lipid-reducing effects of pitavastatin. Serum CETP levels prior to the pitavastatin treatment were positively linked to pitavastatin-induced changes in LDL-C and sd LDL-C levels. Lower basal CETP levels induced greater negative changes in lipid levels, while higher CETP levels caused smaller negative changes. In multivariate analyses including baseline TC and LDL-C levels as independent factors, basal CETP levels were independently and positively associated with changes in LDL-C or sd LDL-C levels. Moreover, pitavastatin decreased serum CETP levels, and these negative percentage changes were strongly and positively associated with the negative percentage changes observed in LDL-C and sd LDL-C. These results suggested that the CETP status during the pitavastatin treatment was markedly associated with the pharmacological effects observed.

The main limitation of our study was that postprandial lipids were measured. Several earlier studies of postprandial lipid changes in healthy subjects showed that levels of LDL-C and sdLDL-C significantly decreased by about 7–9 % at 2–6 h after a meal [19–21]. In diabetic patients, postprandial reduction of LDL-C and sdLDL-C was shown to be about 10 % [22]. However, lipid data variation during 2–6 h after a meal appeared to be much smaller (about 5 %) even in diabetic patients as well as healthy subjects [19–22]. Considering these findings, it seems probably that measurement of postprandial lipid had some degree of influence on the results obtained in our study. It might have affected the measured values positively or negatively depending on the time points of blood collection after a meal.

CETP is a 74-kDa glycoprotein that is synthesized in liver and adipose tissues and subsequently secreted into the bloodstream [1]. Serum CETP levels are known to be up-regulated by a high fat diet for several days and high LDL-C levels, and down-regulated by statin treatments [1, 4, 17, 23]. The expression of CETP was shown to be induced by LXR [5, 6], which was clearly confirmed in our present study with LXR silencing in HepG2 cells (Fig. 3). Since endogenous agonists of LXR are oxysterols, a cholesterol derivative [7], hepatic cholesterol content may be positively related to the expression of CETP, as previously discussed [8]. Atorvastatin, a strong statin, was shown to decrease the expression of LXR-α in macrophages [24]. Pitavastatin repressed LXR activation in a rat hepatoma cell line [25], which was indirectly supported by our result in which the activation of LXR by T0901317 antagonized the pitavastatin-induced reduction in CETP levels in HepG2 cells (Fig. 1). Statin-induced reductions in oxysterol, the endogenous activator of LXR, may lead to a partial reduction in LXR activity [7], and consequently to a reduction in its target genes, CETP and SREBP-1c. These findings implicate the intrahepatic reduction in the mass or activity of LXR in the statin-mediated decrease in serum CETP levels reported in ours and several previous studies [1, 4, 17]. Furthermore, elevated CETP levels in the serum of CETP transgenic mice led not only to an increase in hepatic cholesterol content, but also to the reduced expression of the LDL receptor [26]. Collectively, elevated CETP levels at baseline may reflect an increased hepatic cholesterol content, which may weaken the statin-induced up-regulation of LDL receptor expression, and ultimately attenuate cholesterol-reducing effects. This may explain our result in which lower baseline CETP levels were associated with a greater negative change in LDL-C and sd LDL-C levels.

Statin-modulated LXR expression may be tissue-specific and different among type of statins. Atrovastatin increased expression of LXR-α and LXR-β in HepG2 cells [27] but decreased LXR-α expression in macrophages as mentioned above [24], while in our study pitavastatin had no influence on LXR expression even at 5 μM. Simvastatin increased LXR-α expression in peripheral blood mononuclear cells from diabetic patients with hyperlipidemia [28], but had no effect on the expression in those from non-diabetic hyperlipidemic patients [27]. Therefore, the reduction of LXR expression by statin may occur only under restrictive conditions. Statin-induced reduction of LXR activity may be due largely to reduction of intrahepatic oxy-sterols, endogenous LXR activators.

Pitavastain-induced reduction of SREBP-1c activity is presumed by ApoA-I induction found in our study. Since ApoA-I was previously shown to be up-regulated by liver receptor H-1 (LRH-1) [29], the function of which is suppressed by SREBP-1c [30], pitavastatin may reduce SREBP-1c activity and subsequently enhance LRH-1 function, possibly leading to ApoA-I induction (Fig. 1).

CETP enhances the exchange of CE in LDL as well as HDL for TG in VLDL [2]. This action causes the accumulation of TG in LDL particles as well as HDL particles. In co-operation with CETP, hepatic TG lipase has been shown to induce reductions in the size of LDL via the lipolysis of TG and increases in sd LDL-C levels [2, 13]. In the present study, a positive correlation was not observed between CETP and LDL-C or sd LDL-C at baseline. This may have been due to individual differences in sd LDL-C modifiers such as TG levels themselves and hepatic TG lipase activity according to gene polymorphisms [31, 32]. In contrast, an independent positive association was observed between percentage changes in CETP and those in sd LDL-C because percentage changes in lipids represented the standardized effects of statins in each individual. Furthermore, the pitavastatin-induced suppression of cholesterol synthesis in the liver may be a common pathway to the induction of LDL receptor expression and the reduction in CETP expression. Additionally, pitavastatin-induced reductions in CETP may decrease the rate of sd LDL formation.

Finally, several limitations exist in the present study. Our results were obtained from a small-scale and exploratory study consisting of approximately 50 subjects. Statistical power of paired t-test calculated by a post-hoc method using the data from our study was over 0.9 for pitavastatin-induced reductions in CETP. However, we were not able to calculate the adequate sample size based on reported data, since there are no earlier studies to investigate the influence of CETP status on the lipid-reducing effects of strong stains, similar to ours. Furthermore, it is considered preferable to measure fasting lipid levels and examine HDL particle composition. Therefore, a large-scale study with a more detailed examination of fasting lipid profiles is required to completely define the relationship between the CETP status and lipid-lowering effects of statins.

## Conclusions

The baseline CETP level, a possible surrogate for hepatic cholesterol accumulation, was an independent positive determinant of pitavastatin-induced changes in LDL-C and sd LDL-C levels in hypercholesterolemic patients with type 2 diabetes mellitus. Concurrent and comparable reductions in serum CETP and LDL-C levels suggest that pitavastatin may decrease LXR activity as well as cholesterol synthesis in the liver.

## Methods

### Subjects

The study protocol and informed consent document were reviewed and approved by the Ethics Committees of 6 Hospitals (University of Fukui Hospital, its two affiliated facilities; Fukui Chuo Clinic and Yasukawa Hospital, Tanaka Hospital, Fukui-ken Saiseikai Hospital, and Fukui Prefectural Hospital). This study was registered in UMIN-CTR (ID: UMIN000019020). After being fully informed regarding all aspects of their participation in this study, informed written consent was obtained from all the outpatients. The inclusion criteria were as follows: (1) all subjects had type 2 diabetes mellitus and hypercholesterolemia (LDL-C levels of 120 mg/dL or more, or TC levels of 220 mg/dL or more, at baseline); (2) all subjects were aged between 20 and 80 years; (3) the diabetic condition of all subjects was stable in that the last 2 values of HbA1c were less than 8.4 % (NGSP) with its percentage change being within 10 %. Fifty-three patients who met the inclusion criteria were enrolled in this study. Patients were treated with 2 mg of pitavastatin daily for 3 months. For four patients receiving mild statin (pravastatin, *n* = 3; fluvastatin, *n* = 1), pitavastatin was prescribed in exchange for the mild stain with no washout period. Two patients on fenofibrate were excluded from statistical analyses for investigating the associations of CETP status with lipid-reducing effects of pitavastatin. Dietary and exercise instructions did not change in any patient during the 3-month treatment, and medications that are known to influence lipid metabolism were neither added nor withdrawn. Blood samples were obtained in the post-absorptive state before and after the pitavastatin treatment, and serum was stored at -80 °C until later analysis.

In order to examine postprandial changes in serum CETP levels, blood samples were obtained before and 2 h after breakfast from ten patients with type 2 diabetes mellitus who were admitted to Fukui-ken Saiseikai Hospital and then provided written informed consents, and serum was stored at -80 °C until later analysis.

### Biochemical assays

TC, TG, creatinine (Cr), blood urea nitrogen, and uric acid serum levels and supernatant LDH levels were measured using a standard enzymatic method. The estimated glomerular filtration rate (eGFR) was calculated from serum Cr levels using the new 3-variable Japanese equation [33]. LDL-C and HDL-C serum levels were measured by direct and chemical measurements using commercial kits (Cholestest-LDL and Cholestest-N HDL, respectively; Sekisui Medical, Tokyo, Japan). Sd LDL-C serum levels were measured using a commercially available kit (sdLDL-C “Seiken”, Denka Seiken Co. Ltd., Tokyo, Japan), the details and validation of which were described previously [34]. HbA1c levels were measured by high-performance liquid chromatography. ApoA-I levels were measured by an immunochemistry system using nephelometry (APOA-I AUTO・N “Dai-ichi”, Sekisui Medical). CETP concentrations in the serum were measured using an enzyme-linked immunosorbent assay with monoclonal antibodies (BML, Saitama, Japan). We measured the postprandial serum levels of lipids and proteins for the following reasons: (1) it was difficult to collect fasting blood samples from all patients because approximately 50 % of patients typically underwent a medical examination in the afternoon in the outpatient clinics of the 6 hospitals; (2) a previous study reported that total cholesterol levels were unchanged and HDL-C levels did not change significantly (less than 10 %) in healthy subjects after a meal [19]; (3) we previously reported that no significant difference was observed in serum CETP levels during fasting and 2 h after a meal in healthy subjects [35]; (4) in our pilot study, we observed no significant difference in serum CETP levels before and 2 h after breakfast (2.2 ± 0.70 vs. 2.1 ± 0.61 μg/mL) in patients with type 2 diabetes mellitus (*n* = 10).

### Evaluation of effects of pitavastatin on the expression of genes related to lipid metabolism in the HepG2 cell line

HepG2 cells, a human hepatoma cell line (JCRB1054), were cultured in Dulbecco’s modified Eagle’s medium (DMEM) (Life Technologies Corp., Carlsbad, CA USA) supplemented with high glucose (450 mM), 10 % fetal bovine serum (FBS; Life Technologies Corp.), and 1 % penicillin/streptomycin (Life Technologies Corp.) in a humidified atmosphere containing 5 % CO_2_ and 95 % air at 37 °C. The cells were seeded on 12-well plates at a density of 3 × 10^5^ cells/well in DMEM supplemented with high glucose, 10 % FBS, and antibiotics. Cells grown to semi-confluence were treated for 24 h with DMEM containing T0901317 (Cayman Chemical Co., Michigan, IN, USA; 3 nM to 10 μM), pitavastatin (Kowa Co. Ltd., Nagoya, Japan; 1 to 10 μM), or T0901317 plus pitavastatin. Total RNA was extracted from the HepG2 cells thus treated using a commercial instrument (MFX-2100, Toyobo, Co., Ltd. Osaka, Japan) and commercially available kit (MagExtractor –RNA-, Toyobo, Co., Ltd.). cDNA was synthesized with a High Capacity cDNA Archive kit (Life Technologies Corp.) according to the supplied protocol. TaqMan real-time RT–PCR was performed with a TaqMan ABI 7000 Sequence Detection System (Life Technologies Corp.) using TaqMan Universal PCR Master Mix (Roche Diagnostics K.K., Tokyo, Japan). Unlabeled specific primers and TaqMan MGB probes (6-FAM dye-labelled) from Applied Biosystems were used to detect human CETP (assay ID: Hs00163942_m1), human sterol-regulatory element-binding protein-1c (SREBP-1c; assay ID: Hs01088691_m1), human LXR-α (assay ID: Hs00172885_m1), human LXR-β (assay ID: Hs00173195_m1), and human ApoA-I (assay ID: Hs00985000_m1). A TaqMan human β-actin MGB (VIC dye-labeled) control reagent kit (Applied Biosystems, accession no: NM_001101) was used to detect human β-actin. The CETP, SREBP-1c, LXR-α, LXR–β, ApoA-I, and β-actin cDNA templates were quantified separately using standard curves produced via the serial dilution of standard cDNA. The threshold cycle of each sample was converted to a standard cDNA dilution value (arbitrary units). The cDNA contents of CETP, SREBP-1c, LXR-α, LXR–β, and ApoA-I for each sample were normalized to the level of β-actin, a housekeeping gene, for quantitative analyses. The averaged mRNA amounts of each gene for control cells were set at 1.0.

### Transfection of small interfering RNA against LXR-α or -β

Small interfering RNAs (siRNA) against LXR-α or -β, and control siRNA (a non-targeting siRNA) were purchased from Thermo Scientific Dharmacon (Lafayette, CO, USA). HepG2 cells (30–40 % confluence) were transfected with negative control siRNA or siRNA against LXR-α or -β, at a final concentration of 25 nmol/L using the Lipofectamine RNAiMAX transfection reagent (Invitrogen Corp, Carlsbad, CA, USA), according to the manufacturer’s instructions. After a 48-h incubation, HepG2 cells that had been refreshed with DMEM were treated with or without T0901317 (500 nM) for an additional 24 h and LXR and CETP mRNA expression levels were then analyzed.

### Immunoblot analysis

HepG2 cells were lysed in RIPA buffer containing phosphatase inhibitors (SIGMA-Aldrich, St Louis, MO, USA). Ten micrograms of protein was separated on an 8 % SDS-polyacrylamide gel and then electrophoretically transferred to nitrocellulose membranes (Trans-Blot SD; BioRad, Hercules, CA, USA). The membranes were blocked with Blocking One (Nacalai Tesque, Kyoto, Japan) in Tris-buffered saline (pH 8.0) containing 0.05 % Tween-20 at 37 °C for 1 h and then incubated with a mouse monoclonal antibody against human LXR-α (Cosmo Bio.;Co., Ltd., Tokyo, Japan; 1:200 dilution) or rabbit polyclonal antibody against human LXR-β (SIGMA-Aldrich; 1:2000 dilution) or against human β-actin (Abcam, Cambridge, UK) for 1 h at room temperature. The membranes were then incubated with secondary horseradish peroxidase-conjugated secondary anti-mouse or anti-rabbit antibodies (Dako, Glostrup, Denmark; 1:1000 dilution) at room temperature for 1 h. The secondary antibodies were visualized using the ECL detection system from Thermo Fisher Scientific (Pittsburg, PA, USA). The signal intensities of specific bands were quantified with a densitometer (ImageQuant TL, GE Healthcare UK Ltd, Buckinghamshire, UK).

### Statistical analysis

Continuous variables were expressed as the mean ± SD. The Kolmogorov-Smirnov test proved that all continuous variables fit a normal distribution. Since the reference range of serum CETP levels was not formally defined, several outliers (4.9 μg/mL or more) were ascertained by the Smirnov-Grubbs test and omitted from statistical analyses. Differences in continuous variables between pre- and post-treatment were assessed by the paired *t*-test. Differences in continuous variables between two groups and among three groups were assessed by the Student’s *t*-test and ANOVA with Scheffé’s post-hoc test. The relationship between clinical variables and LDL-C or sd LDL-C levels was examined by Pearson’s analysis or a univariate regression analysis. A *p* value of <0.3 was used as a criteria for selecting a candidate determinant for multivariate regression analysis. A multiple linear regression analysis was performed to identify the independent determinants for LDL –C or sd LDL-C at baseline and their percentage changes. When co-linearity was detected among independent variables (correlation coefficient (r) >0.7), that with the strongest univariate correlation with a dependent variable was selected for the multivariate analysis. When investigating correlations between percentage changes in clinical variables and those in LDL-C or sd LDL-C levels, changes in TC and sd LDL-C or LDL-C, respectively, were excluded from independent variables because they were direct results of the change in LDL-C or sd LDL-C levels. A *p* value of <0.05 (two-tailed tests) was considered significant. All statistical analyses were performed using the JMP system version 5.1.

## Abbreviations

ApoA-I: Apolipoprotein AI
ARBs: Angiotensin II type 1 receptor blockers
CE: Cholesterol ester
CETP: Cholesteryl ester transfer protein
Cr: Creatinine
DMEM: Dulbecco’s modified Eagle’s medium
eGFR: Estimated glomerular filtration rate
FBS: Fetal bovine serum
HbA1c: Hemoglobin A1c
HDL: High density lipoprotein
HMG-CoA: Hydroxylmethylglutaryl-CoA
LDL: Low density lipoprotein
LRH-1: Liver receptor H-1
LXR: Liver X receptor
sd LDL: Small dense low density lipoprotein
siRNA: Small interfering ribonucleic acid
SREBP-1c: Sterol regulatory element-binding protein-1c
TC: Total cholesterol
TG: Triglyceride
VLDL: Very low density lipoprotein
